# Stunting and the hope that must remain; regional and human resource development perspectives; inadequate policy problem identification process in the Tabagsel region of Indonesia

**DOI:** 10.3389/fpubh.2024.1337848

**Published:** 2024-05-27

**Authors:** Arifin Saleh, Rizal Khadafi, Achmad Nurmandi

**Affiliations:** ^1^Social Welfare Study Program, Faculty of Social Science and Political Science, Universitas Muhammadiyah Sumatera Utara, Medan, Indonesia; ^2^Public Administration Study Program, Faculty of Social and Political Science, Universitas Muhammadiyah Sumatera Utara, Medan, Indonesia; ^3^Doctoral Program in Islamic Politics-Political Science, Postgraduate Program, Universitas Muhammadiyah Yogyakarta, Bantul, Indonesia

**Keywords:** stunting, cultural, policy perspectives, Tabagsel region, Indonesia

The post will begin in a slightly unconventional fashion. Two of the three authors were raised in the Southern Tapanuli region (TABAGSEL), which is part of Indonesia's North Sumatra Province. South Tapanuli, Mandailing Natal, Padang Sidempuan, North Padang Lawas, and Padang Lawas are the five current regencies and cities that make up the Tabagsel area. The agricultural, plantation, livestock, and mineral industries, among others, stand to benefit greatly from the region's famed fertile soil and abundant natural resources. The five districts were previously part of South Tapanuli Regency, an administrative unit that existed before the reform era that began in 1998 and ended with the overthrow of the authoritarian Suharto regime. However, in response to calls for greater decentralization of authority and regional autonomy, this Regency was split into four additional Regencies and one Municipality. The combined landmass of the five regencies is about 18,965 square kilometers, or about 25% of North Sumatra Province's total landmass of 72,981 square kilometers. These five districts are home to 1,557,798 people, or 10.31% of North Sumatra Province's total population of 15,115,206 (1).

What does this have to do with the subject of this investigation? Here is a comprehensive explanation. As a local resident, we must acknowledge that it is disheartening to learn that your hometown has the highest prevalence of stunting in North Sumatra Province, and there is a desire to blame the government for its inability to solve the problem. As the director of a survey and research institute, we are aware that blaming the government alone would compromise the researchers' objectivity. We are aware, based on the available data, that the solution to the problem of stunting is not as simple as blaming the government alone. Thirdly, as academics at one of the province's universities, we feel an academic and moral obligation to contribute ideas that can be examined scientifically, impartially, and comprehensively in relation to the various questions raised by this issue. At least these questions remain within the realm of science, particularly in the social sciences: What is the problem, what has been done, and what has been overlooked?

## What is the problem?

The problem stems from the results of the Indonesian Nutrition Status Survey (SSGI) of the Ministry of Health of the Republic of Indonesia, which will be conducted in 2022 and indicate that the prevalence of stunting among children under the age of five in the province of North Sumatra reaches 21.1%. North Sumatra ranks nineteenth among Indonesia's 34 provinces in 2022 with respect to the prevalence of stunting among children under the age of five. North Sumatra is able to decrease the stunting rate among children under five by 4.7 percentage points compared to the previous year. This province will still have a stunting prevalence of 25.8% in 2021 among children under the age of five. Five districts in the Tabagsel region rank among the ten regions in North Sumatra Province with the highest rates of stunting (Table 1).

**Table 1:** Top 10 districts with the highest stunting prevalence of 33 districts/cities in North Sumatra Province, Indonesia.

	**1. Regency/city**	**1. Percentage**
Stunting	1.1. South Tapanuli Regency	39.4%
	1.2. Padang Lawas Regency	35.8%
	1.3. Mandailing Natal District	34.2%
	1.4. Pak Pak Bharat District	30.8%
	1.5. Central Tapanuli Regency	30.5%
	1.6. Humbang Hasundutan District	29.6%
	1.7. Nias Regency	29.4%
	1.8. North Padang Lawas Regency	29.2%
	1.9. Padangsidampuan City	28.8%
	1.10. Dairi District	28.6%

Source: Processed from the 2022 Indonesian Nutrition Status Survey (SSGI) of the Ministry of Health of the Republic of Indonesia (2).

In North Sumatra, 21 districts/cities have a higher prevalence of under-five stunting than the provincial average, while 12 districts/cities have a lower prevalence. In 2022, South Tapanuli district had the highest prevalence of under-five stunting in North Sumatra at 39.4%. This figure increased by 8.6 percentage points from 2021 to 30.8% in 2022. With a prevalence of 35.8%, Padang Lawas Regency ranks second in North Sumatra for the incidence of under-five stunting. Then, Mandailing Natal Regency followed with a prevalence of stunting in toddlers of 34.2%. Labuhan Batu Utara Regency has the lowest prevalence of stunting in North Sumatra at 7.3%, and Medan City, the capital of North Sumatra Province, ranks 27th with a stunting rate of 15.4%.

## What has been done; an approach to policy analysis

In 2017, the Indonesian government unveiled STRATEGI, or the National Strategy for the Acceleration of Stunting Prevention. This project is a comprehensive initiative designed to enhance efforts to prevent stunting, with the goal of constructing a comprehensive framework to effectively address stunting issues. Prioritizing the fields of nutrition, health, education, and social protection is among the measures taken (3). The government has implemented numerous initiatives that prioritize particular nutritional components, such as promoting exclusive breastfeeding, expanding the use of complementary feeding, and distributing vitamin supplements to children and pregnant women. In addition, nutrition-sensitive interventions include efforts to improve WASH (water, sanitation, and hygiene) practices and maternal and child health services (4).

The government also integrates health and nutrition services to provide prenatal, immunization, and other essential health services for mothers and children (5). Increased emphasis on community involvement and awareness campaigns is a further step. Information on nutrition, breastfeeding, and child care practices was disseminated to households with the help of community leaders, health professionals, and volunteers. The government also introduced Direct Cash Assistance Programs (BLT), which provide money to families who are having financial difficulties. One such program is the Family Hope Program (PKH). These initiatives' primary goal is to lessen the effects of poverty by enhancing children's nutrition and education (6). The government also implements nutrition education in educational institutions with the goal of promoting the adoption of healthy eating practices and raising awareness of the significance of consuming a balanced diet.

Additionally, the Government has taken steps to enhance data collection and monitoring systems in order to properly track the development of stunting mitigation and evaluate the effectiveness of interventions (7). The government is also aware of the value of inter-agency cooperation in reducing stunting because this issue calls for effective collaboration and coordination between numerous government departments and agencies. This group effort aims to efficiently address this issue by implementing a thorough strategy spanning several sectors (8). Local government programs in Indonesia have also been adopted strategically to combat the critical issue of stunting in their respective regions. These projects are designed to address the diverse needs and challenges faced by various communities, thereby ensuring a targeted solution (9, 10).

## What's missing; a policy problem identification approach

In the context of the stunting prevention policy in the Tabagsel region, we identified three crucial aspects that have eluded the government's attention at all levels, namely problem identification and inadequate policy analysis. First, the issue is connected to the emancipation of women. In this context, women should ideally play an essential role in terms of nutrition and infant care, and women's empowerment must be increased through education, economic opportunities, and household decision-making authority. Second, in terms of intervention specifics. Taking into consideration local contexts and needs, the intervention in this instance is intended to address various obstacles in overcoming stunting in various regions and communities. The third relates to behavior modification through communication. Communication campaigns for behavior change that are accepted by the community and can generate positive, long-lasting behavior changes are also essential for addressing the issue of stunting.

The preponderance of Tabagsel's ethnic groups are comprised of Angkola, Mandailing, and Batak peoples. In the past, these three ethnic groups adhered to a strong patrilineal system in which males dominated various social aspects. In the context of the cultures and social structures of the Angkola, Mandailing, and Batak ethnic groups, patrilineal elements are present, including:

1) The family name or ancestry is derived from the name of the male ancestor in the father's line. This reflects the patrilineal system, which counts descendants through the line of the father.
2) Inheritance of Property and Status: property and social status are inherited according to paternal lineage. Family property or land ownership may be passed down the patrilineal line to male descendants.
3) Gender Roles: in the traditions of the Angkola, Mandailing, and Batak tribes, the patrilineal system can also reflect gender roles. Men are viewed as family leaders and are responsible for continuing the family lineage.

In practice, the patrilineal system adopted by these three ethnic groups has a significant impact on the pattern of interaction between children and their parents in this region. The interviews we conducted with 250 young fathers in five districts revealed that the majority of men in this region acknowledged that child care is a woman's responsibility. Even though men participate in child rearing and caregiving, this is not a requirement but rather a choice. The logical consequence of this type of social practice is that it adds to the burden of many mothers, because in addition to caring for and caring for their children, the majority of them also work to provide for their families' economic needs, such as farming, trading, private sector employment, and government employment. With some caution, we also assert that the strong tradition of Sunni Islam and the religion practiced by the majority of the population in this region influence and strengthen this condition.

In addition, we discovered that the Lopo/Lapo culture is one of the strong traditions that may have a high correlation with the problem of stunting in this region. Lopo refers to a traditional café that serves a variety of beverages, including coffee, tea, juice, and snacks. This lopo culture can be described simply as a culture of unwinding, chatting with family or friends, watching various television programs, and playing card games, dominoes, chess, or playing the guitar and singing together. Each father spends anywhere between 4 and 7 h per day in this area of the lopo. With specifics for 1–2 h in the morning before work, 1 h during the day, and 3–4 h in the afternoon or evening after returning home. This time coincides with the busy hours of mothers preparing all the family's needs in the morning, including cooking, washing clothes, cleaning the house, preparing for their children's needs, and taking their children to school, before going to work. In the afternoon and evening, the mother's responsibilities remain largely unchanged, but are supplemented by accompanying the child to study, such as helping the child complete schoolwork. Our interviews with 150 pregnant women in this region revealed that they maintained this daily routine from the first week of pregnancy until 3 weeks before the due date. In terms of gender equality, it is evident that women are permitted to work and pursue careers. However, this is not the case when it comes to managing, caring for, and focusing on the growth and development of children. In this regard, women are treated as inferior beings who must bear the heavy burden of conception, childbirth, childrearing, and child education.

This mother's arduous responsibilities lead us to believe that the high stunting rate in the Tabagsel region is caused by a combination of issues, including a divided attention, a tired body, and a husband who spends a lot of time relaxing in traditional cafes. Specifically, we propose that lopo culture must be identified as a problem prior to the government conducting a policy analysis to address the issue of stunting in Tabagsel. In addition, the government must implement detailed interventions and communication campaigns to alter people's behavior. As far as we can tell, no policy has been issued by the federal, provincial, or local governments that addresses this issue. Thus, fathers continue to play a minimal role in raising and caring for their children.

We emphasize the importance of establishing quantifiable measures to identify invisible but long-standing local customs or practices in order to combat stunting. Frequently, the customs and practices of this community can affect the efficacy of policies designed to combat stunting. In many instances, the government disregards cultural aspects of the community and does not involve social science experts who are qualified to design appropriate policies. Therefore, in the future we will emphasize the significance of thoroughly identifying problems for governments in various regions of the world prior to formulating policies. This step reduces the likelihood of employing the incorrect, ineffective, and inefficient strategy to combat stunting.

## Author contributions

AS: Writing – original draft, Resources, Conceptualization. RK: Writing – original draft, Writing – review & editing. AN: Writing – review & editing, Validation, Conceptualization.
